# Evaluation and validation of next-generation sequencing to support lot release for a novel type 2 oral poliovirus vaccine

**DOI:** 10.1016/j.jvacx.2021.100102

**Published:** 2021-06-11

**Authors:** John O. Konz, Tim Schofield, Sarah Carlyle, Rahnuma Wahid, Azeem Ansari, Jeroen R.P.M. Strating, Ming Te Yeh, Hasmik Manukyan, Saskia L. Smits, Erman Tritama, Latri Rahmah, Dori Ugiyadi, Raul Andino, Majid Laassri, Konstantin Chumakov, Andrew Macadam

**Affiliations:** a Center for Vaccine Innovation and Access, PATH, Seattle, WA, United States; b CMC Sciences, LLC, Germantown, MD 20876, United States; c National Institute for Biological Standards and Control (NIBSC), Hertfordshire, United Kingdom; d Viroclinics Biosciences B.V., Rotterdam, the Netherlands; e University of California San Francisco, San Francisco, United States; f Center for Biologics Evaluation and Research, Food and Drug Administration, Silver Spring, MD, United States; g P.T. Bio Farma, Bandung, Indonesia

**Keywords:** Next-generation sequencing, Validation, Poliovirus, OPV, Neurovirulence, cVDPV, circulating vaccine-derived poliovirus, GCV, Geometric coefficient of variation, G: IU, genome to infectious unit ratio, NGS, next-generation sequencing, nOPV2, type 2 novel oral poliovirus vaccine, OPV, oral poliovirus vaccine, SNP, single-nucleotide polymorphism, TNP, tri-nucleotide polymorphism

## Abstract

A novel, genetically-stabilized type 2 oral polio vaccine (nOPV2), developed to assist in the global polio eradication program, was recently the first-ever vaccine granted Emergency Use Listing by the WHO. Lot release tests for this vaccine included—for the first time to our knowledge—the assessment of genetic heterogeneity using next-generation sequencing (NGS). NGS ensures that the genetically-modified regions of the vaccine virus genome remain as designed and that levels of polymorphisms which may impact safety or efficacy are controlled during routine production. The variants present in nOPV2 lots were first assessed for temperature sensitivity and neurovirulence using molecular clones to inform which polymorphisms warranted formal evaluation during lot release. The novel use of NGS as a lot release test required formal validation of the method. Analysis of an nOPV2 lot spiked with the parental Sabin-2 strain enabled performance characteristics of the method to be assessed simultaneously at over 40 positions in the genome. These characteristics included repeatability and intermediate precision of polymorphism measurement, linearity of both spike-induced and nOPV2 lot-specific polymorphisms, and the limit-of-detection of spike-induced polymorphisms. The performance characteristics of the method met pre-defined criteria for 34 spike-induced polymorphic sites and 8 polymorphisms associated with the nOPV2 preparation; these sites collectively spanned most of the viral genome. Finally, the co-location of variants of interest on genomes was evaluated, with implications for the interpretation of NGS discussed.

## Highlights

• Genetic variants were evaluated to assess which were important to ensure nOPV2 quality.
• The cDNA preparation and NGS method was validated through evaluating mixtures of Sabin-2 and nOPV2.
• Pre-specified validation criteria for linearity and precision were met at all positions.
• The method was assessed to be fit-for-purpose for vaccine lot release.
• Understanding the co-location of genetic variants was important to interpret NGS results.

## 1 Introduction

Oral poliovirus vaccines (OPV) made from the Sabin strains have played a critical role in reducing poliomyelitis, including global eradication of the type 2 and 3 wild-type polioviruses [1]. Despite this critical role, improvement of the genetic stability of OPV strains is necessary to reduce or eliminate cases of vaccine-associated paralytic polio, which occur in vaccinees and susceptible contacts at a rate ranging from 2.4 to 9.7 cases per million births in OPV-using countries [2]. In addition, serial transmission of shed vaccine viruses in populations with inadequate herd immunity can lead to their conversion to circulating vaccine-derived poliovirus strains (cVDPV) with a phenotype similar to wild-type viruses. OPV type 2 (OPV2) has a higher tendency to become a cVDPV, accounting for the majority of cVDPV cases in recent years [3]. This tendency has only worsened since the global cessation of routine use of OPV2 in 2016, with 28 countries experiencing cVDPV2 outbreaks in 2019 or 2020 causing over 1300 cVDPV2 cases [4].

The key genetic determinants for the attenuation and reversion of Sabin OPVs are well understood [5]. For OPV2, two sites are primarily responsible for the attenuation: nucleotide 481 in domain V of the 5′ untranslated region and amino acid 143 in viral protein 1 (VP1) [6]. The adenine to guanine reversion at nucleotide 481 (A481G) has been shown to occur within days post vaccination, with almost all vaccinees shedding primarily reverted virus in stool within 2 weeks [7], [8], [9]. In an attempt to control the rate of reversion, regulatory authorities require that Sabin OPV virus seeds and vaccine lots have low levels of domain V reversion (A481G for OPV2) as measured using molecular methods as well as passing neurovirulence tests in either monkeys or transgenic mice [10]. More recent efforts have begun to utilize next-generation sequencing (NGS) as an alternative to the current molecular method which utilizes PCR and restriction enzyme cleavage (MAPREC) [11], [12], [13]. In 2020, the WHO Expert Committee on Biological Standardization reviewed the data on OPV testing by NGS and concluded that it can be used for monitoring manufacturing consistency; however, we are unaware of any vaccine manufacturer formally adopting NGS for routine lot release testing. Interest in NGS for detection of adventitious agents also is high, with both notable progress and challenges reported [14].

Two novel OPV2 strains designed to enhance the stability of the attenuation have been tested in pre-clinical research and three Phase 1 and 2 clinical studies [15], [16], [17], [18], [19]. Data for both strains were encouraging, with nOPV2 candidate 1 [nOPV2-c1] selected for further development. When compared to Sabin mOPV2 (monovalent OPV type 2), the nOPV2-c1 has a similar general clinical safety profile, non-inferior immunogenicity, and promising evidence of enhanced phenotypic stability [19], [20]. The regulators involved in reviewing the Emergency Use Listing application for nOPV2 expressed a strong interest in the manufacturer using NGS to routinely evaluate three nOPV2 quality attributes: (1) preservation of the genetically-modified regions of the virus, (2) levels of genetic variants which might influence neurovirulence or viral fitness, i.e. the safety and efficacy of the vaccine, and (3) absence of contaminating Sabin-2 virus. As such, the manufacturer agreed to adopt NGS as part of the routine quality control of drug substance. We describe here the data leading to selection of three observed polymorphisms for routine evaluation (aim 2) and summarize a method validation study which interrogated specific performance aspects of NGS. These aspects included the limit-of-detection of genetic variants (relevant primarily to aims 1 and 3), and the precision and linearity of variant responses (relevant primarily to aim 2). Additionally, we explore complexities associated with application and interpretation of NGS limit tests to vaccine lots containing sequence heterogeneities.

## 2 Methods

### 2.1 Vaccine samples

The nOPV2 (referred to as nOPV2-c1 or nOPV2.1 in prior publications) lots were generated at P.T. Bio Farma (Bandung, Indonesia) through infection of Vero cells under standard manufacturing production conditions. Differences between nOPV2 and the parental Sabin-2 strain are outlined in Table 1 and include the following: insertion of a new *cis*-acting replication element (cre) in the 5′ untranslated region (UTR), changes in the attenuated domain V of the 5′ UTR to stabilize it against reversion, nucleotide changes made to facilitate construction of the strain, silent changes associated with knocking out the native cre, and changes in the RNA polymerase to improve fidelity and reduce recombination [16]. The titer of the lots was measured by Bio Farma using standard cell culture infectivity (CCID_50_) assay in HEp-2c cells.

**Table 1:** Differences between nOPV2 and Sabin-2 strains. Positions shown are based on nOPV2 numbering. Corresponding position in Sabin-2 reference is 61 nucleotides lower after the cre5 insert.

Region	Position (nOPV2)	Sabin-2	nOPV2
Cre5 insert	121–181	–	61 nt insert
Domain V	530	C	T
	538	C	T
	539	G	T
	542	A	G
	548	G	A
	550	T	C
	551	C	T
	554	G	A
	556	A	C
	561	T	C
	562	G	A
	563	A	G
	565	T	C
	566	G	A
	569	T	C
	588	T	A
	589	G	A
	595	G	A
Molecular biology sites	814	C	A
	817	C	T
	1375	A	T
Cre knockout	4516	C	T
	4519	A	C
	4522	G	A
	4525	C	T
	4528	G	A
	4529	A	T
	4530	G	C
	4543	T	C
Variable site	5587	C	T
3D pol modifications	6159	A	G
	6160	G	A
	6203	G	A
	6205	C	T

Sabin OPV2 reference VC-170180002 P1 was derived at Viroclinics from WHO OPV Reference mOPV2 15/296 (NIBSC). The titer of the stock was measured at Viroclinics using a CCID_50_ in HEp-2c cells.

The genome concentration of lots of nOPV2 and Sabin-2 used in the validation study were measured by quantitative RT-PCR methods specific to the strain. Method details are found in supplementary materials.

Research stocks of nOPV2, Sabin-2 and related strains (molecular clones) used for neurovirulence and temperature sensitivity assays were constructed and recovered at NIBSC through standard methods of mutagenesis, RNA transcription, transfection and amplification as previously described [21], [22].

### 2.2 Whole genome amplification and NGS method

NGS and data analysis were performed on viral RNA isolated from virus samples using a previously-described method [7], [20], [23]. In brief, viral RNA was isolated from 140 µl virus stock using QIAmp Viral RNA mini kit (Qiagen). cDNA preparation and amplification of full-length genome were performed using A7-Sabin2 and U-S7 primers [7]. Tagmentation and library preparation used a Nextera Flex kit (Illumina), followed by 300-cycle paired-end sequencing using a MiSeq reagent kit on a MiSeq instrument with analysis software version 1.8.46 (Illumina) to generate FASTQ files. Data analysis was performed using a proprietary algorithm at Viroclinics comprised of trimming (Trimgalore version 0.4.4), mapping (BWA mem; version 0.7.16a-r1181), and single nucleotide polymorphism (SNP) variant calling at ≥0.5% (for spiking study, 1% for routine use) where multinucleotide polymorphisms and complexes are split into multiple SNPs using the vcfallelicprimitives utility from vcflib library (freebayes; version v1.1.0-60-gc15b070). Supplemental data analyses were performed on some FASTQ files in Geneious Prime software (Biomatters).

### 2.3 Temperature sensitivity assays

Temperature sensitivity of virus growth was assayed by plaque-formation in HEp-2c and Vero cells at different temperatures as described [6]. Temperatures were controlled by incubation of inoculated plates in sealed plastic boxes submerged in water baths whose temperatures fluctuated by <0.01 °C.

### 2.4 Neurovirulence testing in mice

For assessing neurovirulence of vaccine strain and molecular clone viruses, groups of 8 age and sex matched transgenic mice bearing the human poliovirus receptor (Tg66 mice, NIBSC) were inoculated with a range of doses and PD_50_ calculated using the Spearman-Kärber method [16]. Mouse experiments at NIBSC were performed under licenses PPL 80/2478 and PPL 70/8979 granted by the UK Home Office under the Animal (Scientific Procedures) Act 1986 revised 2013 and reviewed by the internal NIBSC Animal Welfare and Ethics Review Board before submission.

### 2.5 Immunogenicity assessment in mice

Immunogenicity in mice was performed as previously described [16]. In brief, groups of ten four-week old, sex-matched mice with the human poliovirus receptor and type I interferon receptor knock-out (PVRTg21/IFNR-ko) were inoculated with varying doses via intraperitoneal injection. Mice in the mock group were administered viral medium. Sera were tested for neutralizing antibody titers using a 100 CCID_50_ challenge dose and assessing cytopathic effect. All procedures were performed in accordance with the guidelines of the Laboratory Animal Center of National Institutes of Health. The Institutional Animal Care and Use Committee of the University of California, San Francisco approved all animal protocols (Approved protocol No. AN128674-01A).

### 2.6 Spiking study

A study involving spiking the Sabin-2 strain into an nOPV2 lot was conducted at Sabin-2 to total infectivity ratios of 0%, 0.5%, 1%, 2%, 5%, 10%, 20% and 100%. Each mixture was prepared independently, and each of the eight stocks was divided into aliquots for later analysis. Three aliquots of each sample were processed through cDNA preparation and NGS as described to yield three results for each of the eight samples. The process then was repeated on a different day on three additional aliquots and using a different MiSeq to generate an additional three results per sample (six total).

### 2.7 Statistical analysis of validation spiking study

The data were reported as the percent frequency of a genetic variant at each of the studied positions. Statistical analyses were conducted using SAS® Version 9.4. Repeatability and intermediate precision were estimated from frequencies generated from each spike level and at each position specified in Table 1 and for ten pre-specified nOPV2 variants. Frequencies were transformed (natural log or ln) for this and other analyses. Repeatability (Rpt) and intermediate precision (IP) were calculated and reported as percent geometric coefficient of variation (%GCV) as described in USP <1033> [24] according to the following formulas:

$$\%GCV_{\mathrm{Rpt}}=100\times \left(e^{\sqrt{{\mathrm{VC}}_{\mathrm{Error}}}}-1\right)\%\;and$$

$${\%GCV}_{\mathrm{IP}}=100\times \left(e^{\sqrt{{\mathrm{VC}}_{\mathrm{Run}}+{\mathrm{VC}}_{\mathrm{Error}}}}-1\right)\%,$$

where ${\mathrm{VC}}_{\mathrm{Run}}$ and ${\mathrm{VC}}_{\mathrm{Error}}$ represent the variability between runs of the genetic sequencing method (n = 2) and the variability among replicates within runs (m = 3 per run) respectively. Note that for values of %GCV less than 20%, %GCV values are nearly identical to the relative standard deviation [25].

Linearity was addressed using analysis of covariance (ANCOVA) on data from each nOPV2 position across spike levels. The nOPV2 position was treated as a categorical variable in the analyses. Mathematical forms of individual frequencies and of Sabin-2 spike levels were treated as the dependent and independent variables, respectively.

For positions representing responses to Sabin-2 spikes frequency was expressed as a proportion while the spikes were re-expressed from infectivity ratios to genome ratios as described in *Translation of spike values infectivity fractions into genomes ratios* below. The natural log transformation was performed on both frequency fraction and genome ratio. Individual slopes were estimated from the linear dose (spike) response relationship at each position for each run. For nOPV2-specific polymorphisms, frequencies were normalized to the 0% (nOPV2-only sample) response and analyzed against the genome ratio.

The limit of detection (LOD) of the NGS method was calculated for selected positions using linear regression of frequency fraction on genome ratio. The estimated slope (b) and intercept (a) from each regression was used together with the standard deviation (s) calculated using the following formulas:

$$\mathrm{yLOD}=a+2\cdot z_{\alpha }\cdot s,$$

$$\mathrm{LOD}=\frac{\mathrm{yLOD}-a}{b},$$

where *yLOD* is the level of response (frequency) corresponding to the LOD, z_α_ is a critical value from the standard normal distribution which is multiplied by 2 to represent the false positive and false negative rates (*α*), *s* is an estimate of the variability of the method which can be taken from the IP estimates at the lowest spike level (0.5%), and *a* and *b* are the estimated intercept and slope from the regression.

## 3 Results

### 3.1 Identifying variants of interest in nOPV2

Mutations known to impact the phenotype of Sabin-2 or which were consistently observed at levels above 5% in nOPV2 preparations were evaluated for fitness in susceptible HEp-2c and Vero cell cultures and a well-accepted transgenic mouse model of neurovirulence through the use of molecular clones. Genetic variants were evaluated in the Sabin-2 and nOPV2 genetic backgrounds. These included G2528A (VP3-E234K), A2969G (VP1-I143V), A3053G (VP1-N171D), and G3425A (VP1-E295K). As shown in Table 2, in both backgrounds, variants in VP1-143 and VP1-171 both increase neurovirulence (i.e. reduce the 50% paralytic dose [PD_50_]); however, in the Sabin-2 background this increase is substantially less than that due to reversion in the primary attenuation site, domain V (A481G). Pairing these two VP1 variants in a double-mutant virus did not further increase virulence.

**Table 2:** Impact of genetic variants on temperature sensitivity and on neurovirulence in transgenic mice. In cases where a 50% paralytic dose was not measurable, the maximum titer inoculated is reported along with the fraction of mice paralyzed at that dose in parentheses. Impact on growth at 37 °C was measured as a reduction in PFU in two cell lines with results falling into three ranges coded as follows: <3-fold (-), 10–100-fold (+), and > 1000-fold (++).

Strain/variant	50% paralytic dose *i.s.*, log CCID50	Impact on growth in culture at 37 °C	
		HEp-2c	Vero
Sabin 2 clone	5.9	–	–
Sabin 2/A481G	1.9	–	–
Sabin 2/VP3-E234K	>5.7 (1/8)	–	–
Sabin 2/VP1-I143V	3.5	–	–
Sabin 2/VP1-N171D	4.1	–	–
Sabin 2/VP1-E295K	>8.1 (3/8)	+	++
Sabin 2/VP1-I143V, VP1-N171D	3.4	–	–
Sabin 2/VP1-N171D, VP1-E295K	6.4	–	–
nOPV2	>8.4 (0/8)	–	–
nOPV2/VP1-E295K	>7.8 (0/8)	+	++
nOPV2/VP3-E234K	>7.6 (0/8)	–	–
nOPV2/VP1-I143V	7.8	–	–
nOPV2/VP1-N171D	7.7	–	–
nOPV2/VP1-N171D, VP1-I143V	>7.9 (2/8)	–	–
nOPV2/VP1-N171D, VP1-E295K	>7.7 (0/8)	–	+

The VP3-E234K variant had no detectable impact on virulence or fitness in cell culture. In contrast, the VP1-E295K variant clearly reduced neurovirulence in the Sabin-2 background with over a 100-fold increase in the estimated PD_50_. A similar reduction of neurovirulence due to VP1-E295K in the nOPV2 strain is expected but was infeasible to demonstrate as nOPV2 already showed no measurable neurovirulence. The reduction in virulence was likely related to a loss of replicative fitness at 37 °C, with a substantially enhanced temperature-sensitive phenotype in both HEp-2c and Vero cells. The fitness impact of VP1-E295K was observed in both Sabin-2 and nOPV2 backbones (Table 2). Interestingly, the combination of the VP1-N171D and VP1-E295K variations in a single virus resulted in phenotypic offset, with levels of neurovirulence similar to the parental strains. This observation was highly relevant to the vaccine lots, as described below.

**Fig. 1:**
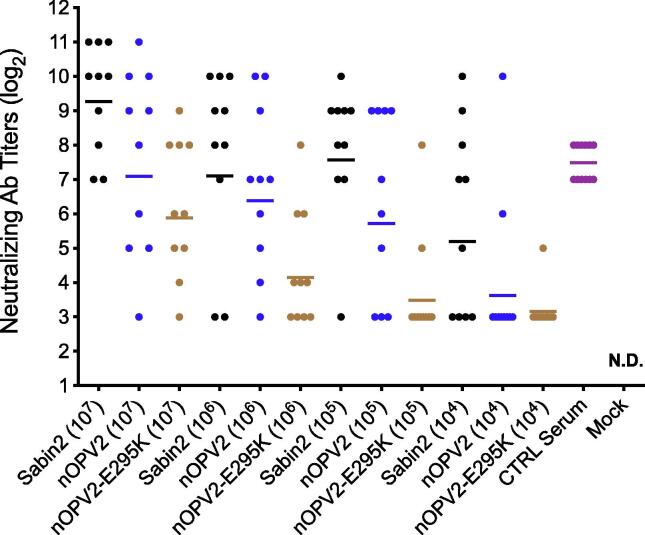
Impact of VP1-E295K variation (at approximately 99% level) on immunogenicity in TgPVR, interferon-receptor knockout juvenile mice. The minimum dilution was 8-fold, with no non-responder mice other than those receiving medium (labeled Mock). Control (CTRL) serum is a positive control for the viral neutralization test. Horizontal segments represent geometric means. ND means not detected (titer < 8).

Because of the impact on replicative fitness at 37 °C in cell culture, an nOPV2 sample with high (*ca.* 99%) VP1-E295K was further assessed for immunogenicity in a permissive mouse model requiring replication for immunogenicity. As shown in Fig. 1, presence of the VP1-295 K variation results in a reduction in immunogenicity versus a strain without the variation, with the dose-response appearing to be shifted by 1–2 logs. Titers were significantly lower for mice administered nOPV2-E295K than nOPV2 at the 10^5^ and 10^6^ dose levels (p = 0.02 for both using Mann Whitney test). Similar results for this variation were observed for nOPV2 candidate 2 (data not shown).

From these observations, three variants in VP1 present in production lots were selected for closer evaluation during routine manufacturing because of their possible impact on immunogenicity (VP1-E295K) or virulence (VP1-I143T and VP1-N171D).

### 3.2 Validation of NGS

Qualification studies covering several of the elements of the NGS workflow individually and together had been conducted over 3 years to ensure the method was fit-for-purpose for analyzing clinical shed virus samples, and particularly for consistently detecting variants (data not shown). The study summarized here focused on extending this work to a more traditional manufacturing assay validation which involved understanding the precision of variant calling in an nOPV2 lot, a pure Sabin-2 sample, and varying levels of Sabin-2 spiked into the nOPV2 lot. The linearity of the spike response for the Sabin-specific and nOPV2-specific variants and the limit-of-detection for the Sabin-specific variants were also evaluated.

#### 3.2.1 Impact of bioinformatics approach

**Fig. 2:**
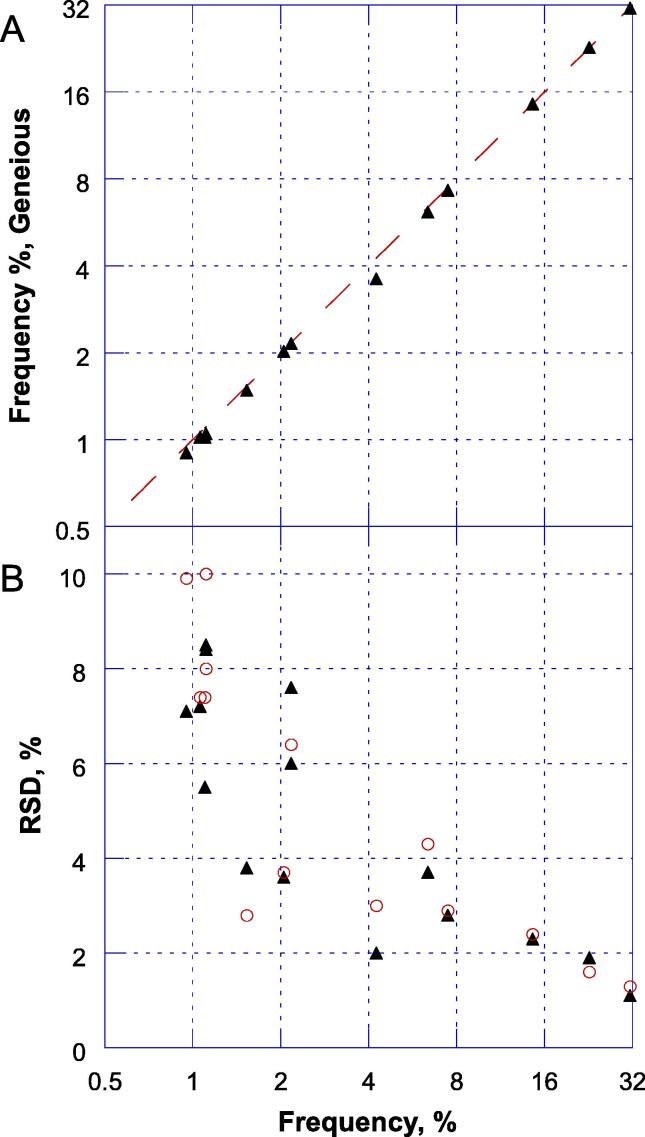
Concordance between standard bioinformatics workflow and Geneious prime mapping on frequency and variability of SNP determination for an nOPV2 lot. (A) Average frequencies measured for the 14 SNPs (y = x line is shown for reference). (B) Relative standard deviations for the SNPs as a function of average frequency. Standard analyses are shown as triangles and Geneious analyses as circles.

The robustness of the NGS procedure to mapping and variant calling software was informally explored by comparing the validated custom pipeline to Geneious prime software when analyzing common FASTQ files. Fig. 2a shows the high concordance between the results for nOPV2 for 14 SNPs that appeared at above 1%. Additionally, the variability between the replicates for the SNPs was similar, with an inverse relationship between the relative standard deviation and average frequency of the SNP (Fig. 2b).

#### 3.2.2 Translation of Sabin spike infectivity ratios into genomes ratios

**Fig. 3:**
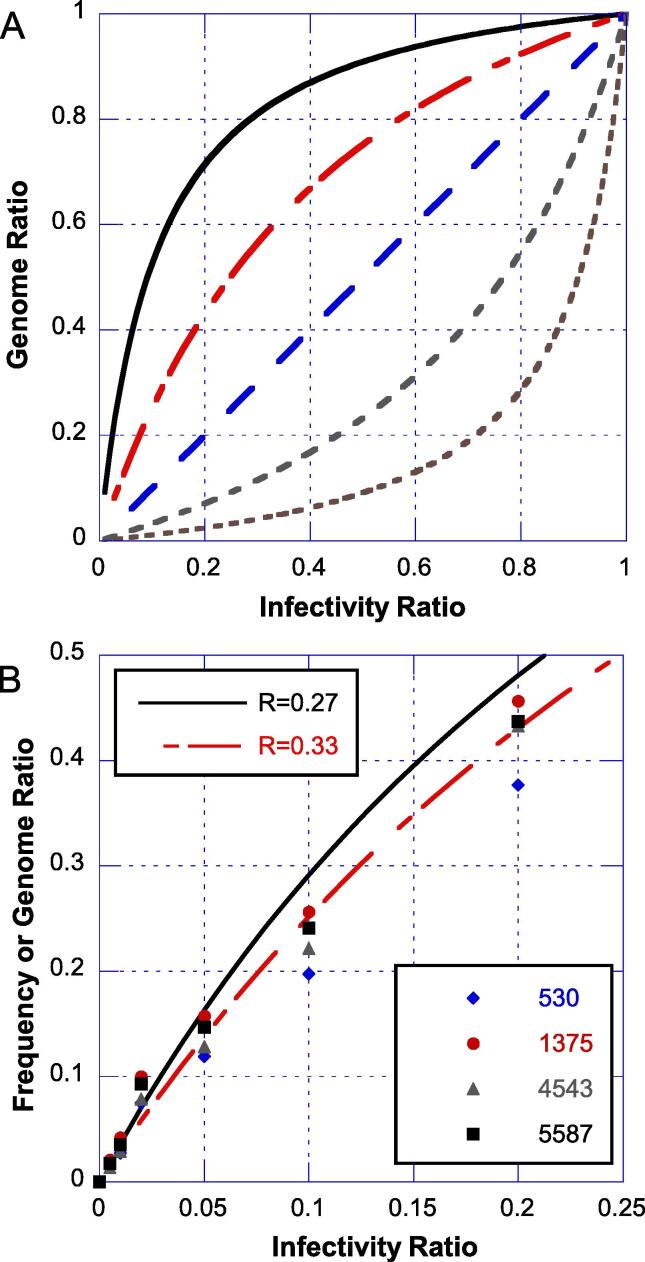
Impact of differences between genome-to-infectious unit ratio of viral strains on NGS frequency responses. (A) Theoretical relationship between infectivity ratio and genome ratios are shown for virus pairs with ratios of genome-to-IU ratios of 0.1, 0.33, 1, 3.3, and 10. (B) NGS frequencies at selected Sabin-specific nucleotides plotted against the spike infectivity fraction. Calculated values of the genome ratio based on NGS fitting and RT-PCR measurements of the pure stocks are overlaid as curves.

Because of the urgency caused by the ongoing cVDPV2 outbreaks, the spiking study was initiated before a method to directly measure genome concentrations in viral preparations was available. Spikes of Sabin-2 into the nOPV2 preparation were prepared using infectivity measurements, targeting specific ratios of Sabin-2 to total infectivity. Given that NGS measures genomes, the infectivity ratio and NGS frequency are not anticipated to be linear if the two virus strains have different genome-to-infectious-unit (G:IU) ratios. Specifically, the genome ratio for two viruses can be expressed as

$$\mathrm{GR}=\frac{1}{\left[1+R\left(\frac{1}{\mathrm{IR}}-1\right)\right]}$$

where GR is the genome ratio, IR is the infectivity ratio, and R is the ratio of the G:IU ratios of the two strains. Exemplary plots for varying R are shown in Fig. 3a.

As anticipated, a non-linear response was observed for this study in comparing the infectivity ratio with the NGS responses for a subset of the Sabin-specific positions (Fig. 3b). To address this issue, the constant R was fit through error minimization from the infectivity ratio versus NGS frequency relationship for four spike-induced SNPs not associated with the genetic stabilization (A814C, T817C, T1375A, and T5587C). Calculated genome ratios were then used for subsequent data analyses. After the validation exercise had been completed, an RT-PCR method was used to independently measure the genome concentration of each pure virus stock (n = 6 each), allowing R to be independently estimated. The calculated value of R using RT-PCR was consistent with the fit value from NGS (0.27 versus 0.33, respectively). Estimated genome ratios for the two R estimates are overlaid in Fig. 3b.

We noted during these preliminary assessments that measured frequencies were lower in locations where Sabin-specific positions were clustered on the genome. The cause of this observation is evaluated in the supplementary materials.

#### 3.2.3 Repeatability and intermediate precision assessment across genome

According to ICH Q2 [26], the precision of an analytical method should be considered at multiple levels. Repeatability, or intra-assay variability, reflects variability under the same conditions over a short interval of time while intermediate precision incorporates within-lab variability associated with different assay days, analysts, and equipment. For the spiking study, the three replicates within each day provided information on repeatability while the evaluation of the overall dataset (three replicates times two runs) provided an estimate of intermediate precision.

**Fig. 4:**
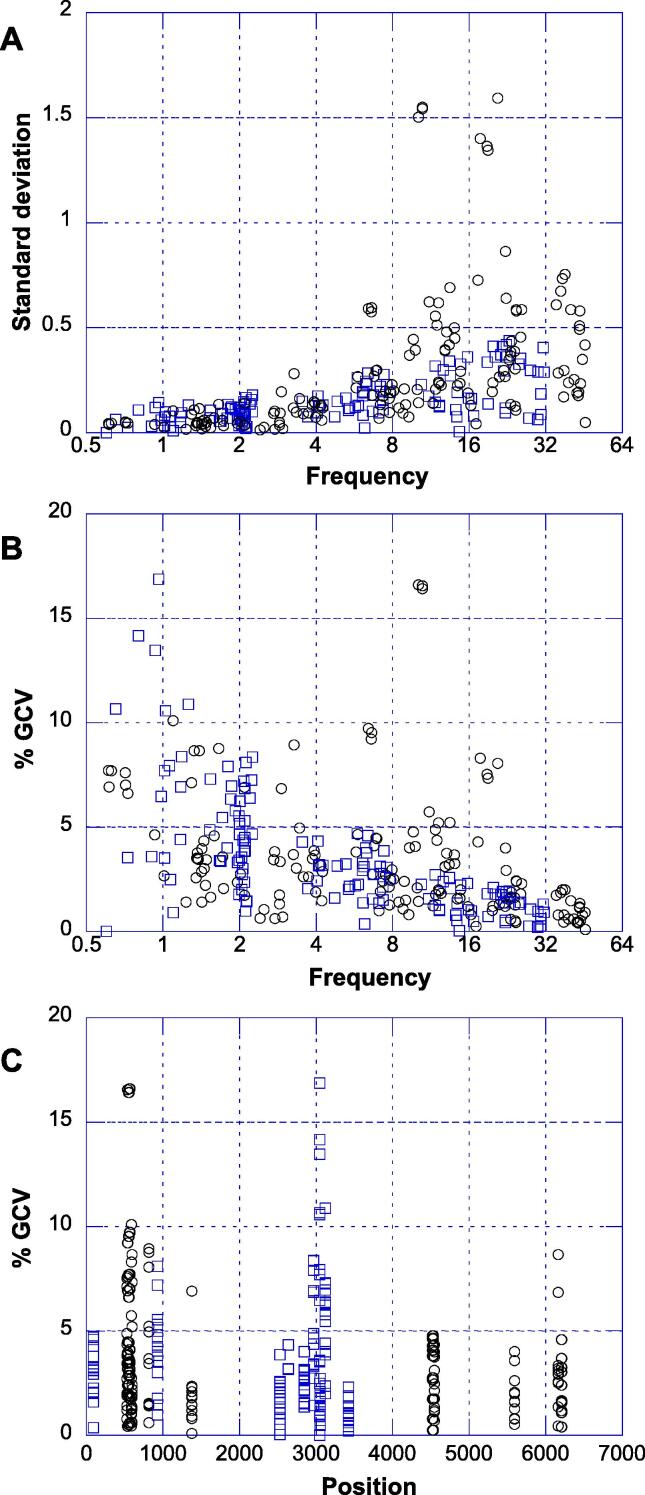
Variability of measurements within runs. Each data point corresponds to n = 3 measurements of a variation at a single spike level. Sabin-spike induced variants are shown black circles, nOPV2 variants as blue squares. (A) Standard deviation of the frequency as a function of the mean frequency (both in percent). (B) Percent Geometric Coefficient of Variation (%GCV) as a function of the mean frequency measurement in percent. (C) %GCV as a function of position in the nOPV2 genome.

Fig. 4 shows an exploratory analysis of the full dataset including 34 Sabin-2-spike-specific locations and 10 nOPV2-specific variants. The absolute variability (standard deviation) tended to increase with mean frequency measurement while the relative variability (percent geometric coefficient of variation or %GCV) generally was inversely related to frequency. No obvious impact of genome position was observed.

Repeatability and intermediate precision were calculated at all combinations of genome location and spike level. Both intra-run and inter-run variance component estimates were determined. In general, repeatability contributed the majority of variance with a median contribution of over 85% of the total. (A histogram of the repeatability portion of the error is provided as Supplemental Fig. 2.) Prospective validation acceptance criteria for repeatability (<15% GCV) and intermediate precision (<20% GCV) were met at all spike locations and levels analyzed with ranges of 0.5–11.6% and 0.6–15.7%, respectively. Justification for the acceptance criteria is described in the supplementary material.

Based on formal statistical analysis for the three nOPV2 SNPs of particular interest—T2970C, A3053G, and G3425A—the intermediate precision expressed as a %GCV ranged from 3.1–8.1%, 0.8–3.1%, and 0.4–1.7% respectively across the seven samples containing nOPV2.

#### 3.2.4 Evaluation of linearity of spike responses

For quantitative lot release tests, establishing linearity of measurements over a relevant range is required. A formal linearity assessment was performed using regression analysis on frequency fraction and the spike genome ratio (both natural log scaled) for each of the 34 Sabin-specific variants and eight nOPV2-specific SNPs. Measurement of the absence of the cre5 (deletion starting at nucleotide 121) also was analyzed informally despite variants not meeting the required quality score for formal variant calling. For the nOPV2-specific SNPs, ten positions had been pre-specified for evaluation based on being observed consistently in production lots. Of these, two were eliminated from this evaluation because they were also present in similar amounts in the Sabin lot used for the spike, leading to an absence of a meaningful spike response.

For the nOPV2-specific SNPs, frequencies were re-normalized to a 0 to 1 scale through the normalization 1 – F/F_O_ where F is the measured frequency and F_O_ is the mean frequency of the six pure nOPV2 replicates (0% spike) at the relevant position.

**Fig. 5:**
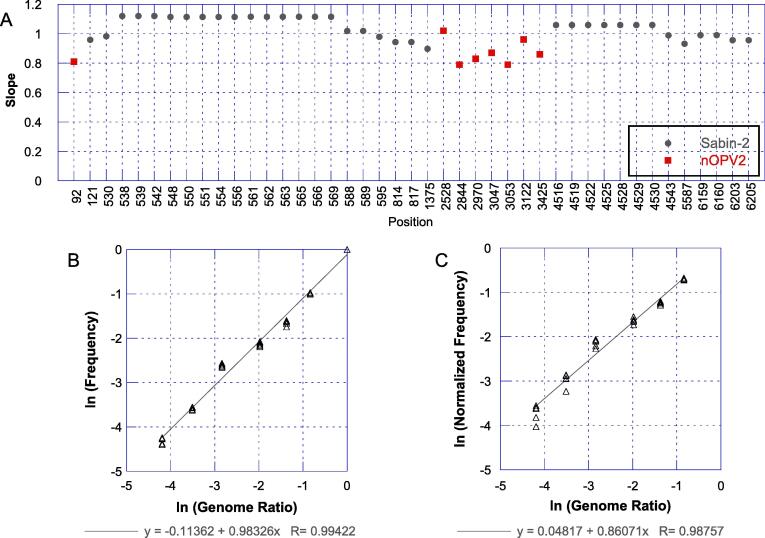
Assessment of linearity of spike responses. (A) Estimated slope of natural log of frequency as a function of the natural log of spike genome fraction for all Sabin-specific and nOPV2-specific variants. Exemplary plots are shown for a Sabin-spike induced SNP (T530C, panel B) and an nOPV2-specific SNP (G3425A, panel C).

Fig. 5 shows the slopes of the log–log regression analyses for the Sabin-specific variants and nOPV2-specific variants, where the slope approximates the exponent (n) of the relationship

$$y=m\times x^{n}+b$$

in cases where the intercept b (the background frequency) is small relative to the measurements. Therefore, for linear relationships, the slope of the log–log plot has an expected value of 1. The slopes of the regressions ranged from 0.90 to 1.12 for the 35 Sabin-spike variants, and 0.79 to 1.02 for the nOPV2-specific variants diluted by the spiking. Exemplary plots for two positions are shown in Fig. 5b and c. These results demonstrate that the frequency responses are linear (i.e., individual slopes meet the acceptance criterion for linearity, between 0.5 and 1.5) across the genome.

**Fig. 6:**
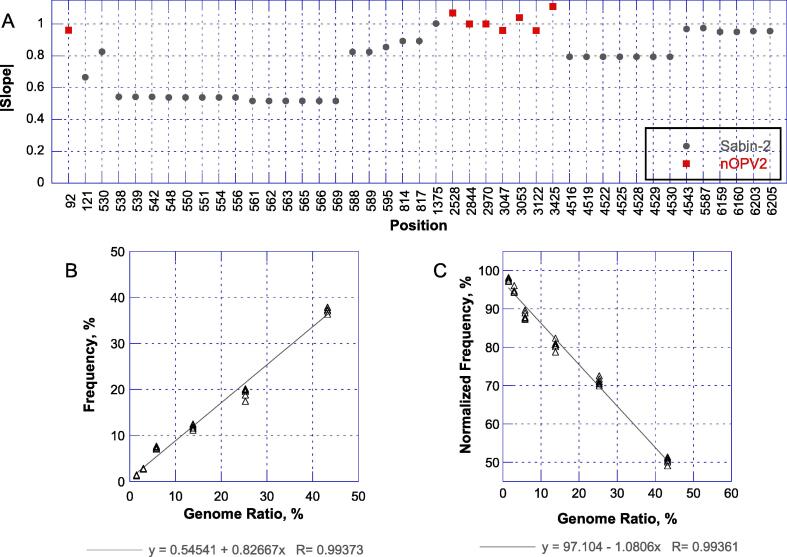
Assessment of slopes of spike responses. (A) The slope of frequency versus spike genome ratio relationship for Sabin-spike-induced polymorphisms and for variants present in nOPV2. Note that for the nOPV2 variants, the slopes are dilution responses and therefore negative. Exemplary plots are shown for a Sabin-spike induced SNP (T530C, panel B) and and nOPV2-specific SNP (G3425A encoding VP1-E295K, panel C).

While not part of the formal acceptance criteria for the method, we also estimated the slopes of the regression of frequency (normalized frequency for nOPV2-specific positions) against spike genome percentage. These values are shown in Fig. 6a, with exemplary plots for two SNPs in Fig. 6b and 6c. In this case, the expected slope value is 1 for the Sabin-specific SNPs and −1 for dilution of the nOPV2-specific SNPs.

For the variants associated with Sabin-2 spiking, the slopes ranged from 0.52 to 1.00. Values with lower slopes are those impacted by clustering on reads as described in the supplementary materials, such as the interior domain V sites (i.e. 538 to 569). The clustering on reads also made these slope evaluations non-independent, as apparent by the identical slopes for sets of nearby positions. For the non- and less-impacted positions, the slopes approached the expected value of 1. For example, for positions 1375, 4543, 5587 and 6205 the slopes were all between 0.95 and 1.00. The nOPV2-specific SNPs all showed slopes near −1 with a range of −0.96 to −1.11.

#### 3.2.5 Limit-of-detection of variant calling

Using a subset of the Sabin-specific positions that were unimpacted or minimally impacted by the clustering artifact (530, 588, 589, 595, 814, 817, 1375, 4543, 5587, 6159, 6160, 6203, and 6205), limit-of-detection (LOD) values were calculated as described in Methods for exclusion of greater than 5% false positive or false negative rates. LODs ranged from 0.38% to 0.47% for these 13 sites. Reducing the acceptable false positive or false negative rate to 1% led to LODs ranging from 0.54% to 0.66%. These values are below the reporting threshold (≥1%) thus signifying the validity of positive responses in the assay.

These results were supported by an analysis confirming that each of the 34 nucleotide differences between Sabin-2 and nOPV2 (Table 1) were identified as variants in all replicates at all spike levels (1224/1224 assessments). The absence of the cre5 also was consistently detected but with quality scores ranging from 25 to 27 which did not meet the threshold (Q > 30) for variant calling. Conversely, the Sabin-2 variants were negative (<0.5%) in all assessments for the pure nOPV2 sample (0/210). Additionally, ten positions (113, 200, 502, 540, 620, 1961, 3025, 4499, 6146, 7000) near the nOPV2 modified sites and without detected variants in either pure sample were negative in all 6 replicates of the 6 spiked samples for a 0/360 false-positive rate.

### 3.3 Variants of interest and their co-location on genomes

The close proximity of the three SNPs of interest in VP1 coupled with the use of 300-cycle NGS allowed us to assess SNP co-location on genomes in a characterization effort unassociated with the formal method validation or lot release efforts. Fifteen manufacturing lots were analyzed. In this work, paired reads from the NGS were merged to extend lengths to up to 563 nucleotides. The merged reads were then mapped against shorter reference sequences containing two of the variants of interest (nucleotides 2969–3054 and 3052–3426) requiring that mapped reads cover the full sequence. Coverage exceeded 7000 reads in all cases for the sequence containing 2970 and 3053 and ranged from 113 to 1515 reads for the sequence containing 3053 and 3425. Variant frequency estimates from these analyses compared to the standard bioinformatics showed only modest differences, suggesting that the subset of reads mapped was representative (see supplementary materials for details, including an in-depth analysis of the nOPV2 lot used in the validation study).

**Fig. 7:**
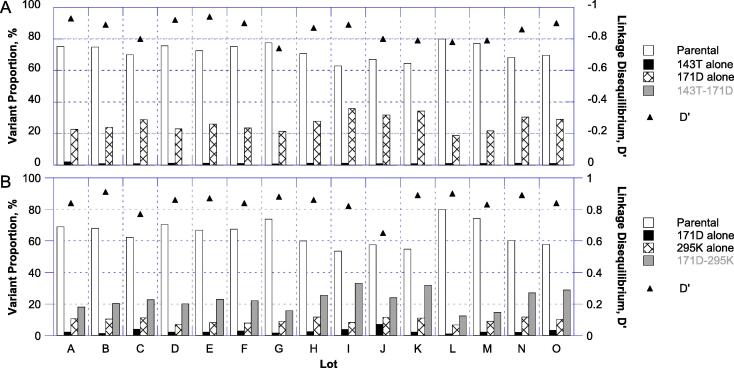
Assessment of co-location of pairs of variants from fifteen nOPV2 lots. (A) Proportions of the four combinations of parental and variant nucleotide at positions 2970 (VP1-143) and 3053 (VP1-171) derived from reads which map to the full region from 2969 to 3054. Parental nucleotides are 2970T (VP1-143I) and 3053A (VP1-171N) and variant are 2970C (VP1-143T) and 3053G (VP1-171D). (B) Proportions of the four combinations of parental and variant nucleotide at positions 3053 (VP1-171) and 3425 (VP1-295) derived from reads which map to the full region from 3052 to 3426 are shown. Parental nucleotides are 3053A (VP1-171 N) and 3425G (VP1-295E) and variant are 3053G (VP1-171D) and 3425A (VP1-295 K). In both panels, linkage disequilibrium values (D’) are shown as triangles.

These analyses showed that the 2970C SNP coding for VP1-143T and the 3053G SNP coding for VP1-171D were generally seen individually, with co-location exceedingly rare (Fig. 7a). The analysis of the 3425A SNP (coding for VP1-295K) with 3053G showed the opposite behavior, with co-location being much more common than would be predicted if random (Fig. 7b). The non-randomness is exemplified in the linkage disequilibrium values (D′), which are 0 for unlinked variations, −1 for negatively linked, and +1 for positively linked. On average, 89% (range 77–94%) of the VP1-171D variant was found in genomes which also contained VP1-295K. The preferred co-location of VP1-171D and VP1-295K on genomes was of particular interest since it has three implications: (1) the fraction of viruses with parental 171N and 295E together is higher than would be expected if randomly distributed and much higher than if co-location did not occur at all, (2) only a small fraction (11% on average) of the VP1-171D-containing viruses potentially have increased virulence, and (3) only a minority of the VP1-295K-containing viruses potentially have a fitness defect. (Points 2 and 3 are demonstrated in Table 2.) For example, for lot I from Fig. 7b standard NGS reports 171D and 295K frequencies of 34% and 43%, respectively, while the co-location analysis clarifies that only 4% of the virus potentially has increased virulence (171D-295E), 8% potentially has reduced fitness (171N-295K), and the balance has parental-like attributes (54% 171N-295E and 33% 171D-295K).

## 4 Discussion

For nOPV2, the whole genome amplification and NGS assay allows the simultaneous assessment of three different attributes of nOPV2 quality that were of interest to regulatory authorities: (1) preservation of the genetically modified regions of the virus, (2) confirmation of identity including absence of Sabin-2 contamination, and (3) limit testing for variants which may have clinical significance. The first and second of these were addressed through specifications ensuring that the modified regions of the virus (e.g. domain V from nucleotide 529–596) do not have detected polymorphisms (above a 1% reporting threshold), noting that for the second objective Sabin-strain contamination would also appear as variants of the modified nucleotides. The third objective required the assay to be used in a quantitative capacity to compare variant levels in commercial lots to levels in clinical trial lots for three variants of interest.

Because of the large number of nucleotides (>150) involved in the specifications, evaluation of the performance characteristics of variant identification and measurement individually by site using molecular viral clones was clearly infeasible. Instead, we utilized a more general approach that involved spiking the parental Sabin-2 strain into the nOPV2 strain, which allowed for the evaluation of all 35 differences between the strains in a single study; limitations associated with this approach are discussed below.

Overall, the performance characteristics of the NGS method met expectations for its intended uses. The method reliably detected all polymorphisms introduced through spiking and did not detect polymorphisms in 10 positions chosen as negative controls. The intermediate precision (%GCV) of the method was below 20% for all 44 locations evaluated at all spike levels which met the method suitability requirements as defined by the clinically-linked specifications on positions 3053 and 3425 and was generally below 10%. The precision was consistent with previous reports for detection of domain V reversion in Sabin-3 using a similar method [11]. Precision was similar across the genome at a particular SNP frequency, and relative variability (%GCV) generally declined as the frequency of the SNP increased. This observation also was noted for the 2493U SNP in Sabin-3 during a collaborative study comparing NGS to MAPREC [13].

Linear spike-responses (i.e., directly proportional changes in response with change in level) are required for quantitative release tests and were observed across the genome. This observation held for both the dose response of polymorphisms associated with the spike as well as the dilution response in the nOPV2-specific polymorphisms. The consistency of the linearity and precision suggests that other positions between those assessed that were not explicitly evaluated in this validation study are also likely to be well behaved. The limit-of-detection of SNPs in the method varied from 0.54 to 0.66% for false positive/negative rates of 1% supporting the routine reporting threshold of 1% used in the method.

Based on literature and this work, three of the SNPs consistently present in nOPV2 lots were potentially of interest in terms of virulence or viral fitness. Variations in VP1-143 are well-known as the secondary locus for attenuation of Sabin-2 [6], [9] and one (VP1-I143T) was consistently detected at low levels. The VP1-N171D variant had previously been observed, albeit infrequently, in vaccine-derived isolates [27] but had not previously been shown to increase neurovirulence. The third variant, VP1-E295K, resides in the receptor-binding footprint of the virus [28]. We demonstrated that VP1-E295K increases temperature sensitivity in cell culture and reduces immunogenicity in permissive mice, presenting a potential concern related to immunogenicity in humans if levels of the parental VP1-295E were too low. Interestingly, for the primary lot in this study and other manufacturing lots, the SNPs encoding the VP1-N171D and VP1-E295K variants were generally co-located on genomes. We showed that these variants phenotypically offset one another when co-located (Table 2) considering both neurovirulence and temperature sensitivity. This observation significantly mitigates concern over their presence in vaccine lots since the level of virus with reference nucleotides in the two positions is in fact higher than would be estimated if randomly distributed, and because the fraction with both variants is of limited concern. Supporting the latter point is additional evidence from the Phase 2 clinical trial, where nOPV2 administration was shown to be generally safe, well-tolerated, and immunogenic [19]. NGS data on fecally-shed vaccine virus from the trial of nOPV2 showed frequent selection of virus with both VP1-295K and VP1-171D *in vivo* (unpublished data, manuscript in preparation), proving that it does not suffer a significant fitness defect. This example highlights one of the risks associated with application and interpretation of NGS—that co-location on genomes may be important to interpretation of results but is often not feasible to assess, and rarely assessed even when feasible due to the unique bioinformatics required.

Nonetheless, out of caution and considering requests from the regulatory reviewers, the manufacturer incorporated specifications for lot release to ensure that the amount of the vaccine virus containing the two virulence-increasing variants (VP1-143T and VP1-171D) would remain below the maximum level studied clinically and that the amount of the parental VP1-295E-containing virus would remain above the level in the low dosage lot used to establish non-inferiority of the immune response.

An initial limitation of the spiking study was the use of infectivity measurements to generate mixtures. This approach required the ratio of the genome-to-infectious-unit (G:IU) ratios of the two strains to be estimated using the curvature of the NGS frequency versus infectivity plot. This ratio then allowed genome ratios to be calculated from the infectivity spike fractions. The validity of this approach was confirmed when genome concentrations of the preparations were measured with RT-PCR. To our knowledge, use of NGS to estimate the ratio of G:IU ratios between related strains has not been previously described and could be of broader utility when quantitative RT-PCRs are not readily available.

Another limitation of the spiking study was underestimation of spike-induced polymorphisms in regions of the viruses with a high density of differences between nOPV2 and Sabin-2 i.e. domain V and, to a lesser extent, the cre knockout. This bioinformatic artifact was not relevant to routine use of the assay as such dense clusters of SNPs are not present in nOPV2 lots and would not be encountered in future lots derived from the well-characterized, tiered virus seed system. Future validation studies should ideally segregate the objectives of understanding the ability to detect a Sabin-2 contamination from understanding the ability to detect polymorphisms in the product, avoiding spike viruses with locally high densities of substitutions (compared to the reference virus) for the latter objective.

To our knowledge, this is the first use of NGS during routine lot release for a vaccine. While appropriate in the context of utilizing a novel vaccine under an Emergency Use Listing, the complexities of executing the NGS method in a GMP environment and maintaining the method in a validated state long term are numerous. While detailing these complexities is outside the scope of this work, examples of the challenges include that method execution is highly complex with many different biochemical and computational steps and that the method requires a considerable number of reagents, many biological in nature, which are not in the direct control of the manufacturer. As such, and despite the excellent performance shown herein, it should be considered if such use as a release test should be time-limited. For example, the method could be transitioned from routine release to periodic and event-driven characterization (e.g. when introducing a new working virus seed lot or process change). Another possibility could be to continue use of NGS after manufacturing consistency is established as an alternative to routine animal neurovirulence testing of each lot. Factors in this decision should include longer term trending of the nOPV2 production process consistency and understanding if rare clinical risks associated with OPV evolution occur during nOPV2 deployments.

In conclusion, the reverse-transcription, whole-genome amplification and NGS method appears to be fit-for-purpose for the evaluations under consideration during nOPV2 lot release, including ensuring preservation of the nOPV2-specific modifications, detection of Sabin-2 contamination, and limit testing on three variants with potential clinical impact.

## Declaration of Competing Interest

The authors declare the following financial interests/personal relationships which may be considered as potential competing interests: ET, LR, and DU are employed by P.T. Bio Farma, the manufacturer of the novel OPV2. JRPMS and SLS are employees of Viroclinics Biosciences, which is paid by the vaccine manufacturer and PATH for performing the NGS analyses.

## References

[1] World Health Organization (WHO). Report from the Twentieith Meeting of the Global Commission for the Certification of the Eradication of Poliomyelitis. Geneva; 2019.

[2] Platt L.R., Estivariz C.F., Sutter R.W. (2014). Vaccine-associated paralytic poliomyelitis: a review of the epidemiology and estimation of the global burden. J Infect Dis.

[3] Alleman M.M., Jorba J., Greene S.A., Diop O.M., Iber J., Tallis G. (2020). Update on vaccine-derived poliovirus outbreaks — worldwide, July 2019–February 2020. MMWR Morb Mortal Wkly Rep.

[4] GPEI. Circulating vaccine-derived poliovirus n.d. https://polioeradication.org/polio-today/polio-now/this-week/circulating-vaccine-derived-poliovirus/ [accessed February 22, 2021].

[5] Sutter RW, Kew OM, Cochi SL, Aylward RB. Poliovirus vaccine--live. In: Plotkin SA, Orenstein WA, Offit PA, editors. Vaccines. 6th ed. Elsevier; 1993. p. 598–645.

[6] Macadam A.J., Pollard S.R., Ferguson G., Skuce R., Wood D., Almond J.W. (1993). Genetic basis of attenuation of the Sabin type 2 vaccine strain of poliovirus in primates. Virology.

[7] Laassri M., Dragunsky E., Enterline J., Eremeeva T., Ivanova O., Lottenbach K. (2005). Genomic analysis of vaccine-derived poliovirus strains in stool specimens by combination of full-length PCR and oligonucleotide microarray hybridization. J Clin Microbiol.

[8] Dunn G., Begg N.T., Cammack N., Minor P.D. (1990). Virus excretion and mutation by infants following primary vaccination with live oral poliovaccine from two sources. J Med Virol.

[9] Stern A., Te Yeh M., Zinger T., Smith M., Wright C., Ling G. (2017). The Evolutionary Pathway to Virulence of an RNA Virus. Cell.

[10] World Health Organization (WHO). WHO Technical Report Series, No. 980 Annex 2 Recommendations to assure the quality, safety and efficacy of poliomyelitis vaccines (oral, live, attenuated); 2014.

[11] Sarcey E., Serres A., Tindy F., Chareyre A., Ng S., Nicolas M. (2017). Quantifying low-frequency revertants in oral poliovirus vaccine using next generation sequencing. J Virol Methods.

[12] Deng Y., Cai W., Li J., Li Y., Yang X., Ma Y. (2019). Evaluation of the genetic stability of Sabin strains and the consistency of inactivated poliomyelitis vaccine made from Sabin strains using direct deep-sequencing. Vaccine.

[13] Charlton B., Hockley J., Laassri M., Wilton T., Crawt L., Preston M. (2020). The use of next-generation sequencing for the quality control of live-attenuated polio vaccines. J Infect Dis.

[14] Khan AS, Blümel J, Deforce D, Gruber MF, Jungbäck C, Knezevic I, et al. Report of the second international conference on next generation sequencing for adventitious virus detection in biologics for humans and animals. Biologicals, vol. 67. Academic Press; 2020. p. 94–111. https://doi.org/10.1016/j.biologicals.2020.06.002. 10.1016/j.biologicals.2020.06.002 PMC7351673 32660862

[15] Konopka-Anstadt J.L., Campagnoli R., Vincent A., Shaw J., Wei L., Wynn N.T. (2020). Development of a new oral poliovirus vaccine for the eradication end game using codon deoptimization. Npj Vaccines.

[16] Yeh M.T., Bujaki E., Dolan P.T., Smith M., Wahid R., Konz J. (2020). Engineering the live-attenuated polio vaccine to prevent reversion to virulence. Cell Host Microbe.

[17] Van Damme P., De Coster I., Bandyopadhyay A.S., Revets H., Withanage K., De Smedt P. (2019). The safety and immunogenicity of two novel live attenuated monovalent (serotype 2) oral poliovirus vaccines in healthy adults: a double-blind, single-centre phase 1 study. Lancet.

[18] De Coster I., Leroux-Roels I., Bandyopadhyay A.S., Gast C., Withanage K., Steenackers K. (2021). Safety and immunogenicity of two novel type 2 oral poliovirus vaccine candidates compared with a monovalent type 2 oral poliovirus vaccine in healthy adults: two clinical trials. Lancet.

[19] Sáez-Llorens X., Bandyopadhyay A.S., Gast C., De Leon T., DeAntonio R., Jimeno J. (2021). Safety and immunogenicity of two novel type 2 oral poliovirus vaccine candidates compared with a monovalent type 2 oral poliovirus vaccine in children and infants: two clinical trials. Lancet.

[20] Wahid R., Mercer L., Macadam, A., Carlyle, S., Stephens, L., Martin, J., et al. Assessment of genetic changes and neurovirulence of shed Sabin and novel type 2 oral polio vaccine viruses. npj Vaccines 2021 [in press]. 10.1038/s41541-021-00355-y PMC8322168 34326330

[21] Burrill CP, Strings VR, Andino R. Poliovirus: generation, quantification, propagation, purification, and storage. Curr Protoc Microbiol 2013;29:15H.1.1-15H.1.27. https://doi.org/10.1002/9780471729259.mc15h01s29. 10.1002/9780471729259.mc15h01s29 PMC4826561 23686830

[22] Burrill CP, Strings VR, Schulte MB, Andino R. Poliovirus: generation and characterization of mutants. Curr Protoc Microbiol 2013;Chapter 15:Unit 15H.2. https://doi.org/10.1002/9780471729259.mc15h02s29. 10.1002/9780471729259.mc15h02s29 PMC4826568 23686829

[23] Neverov A., Chumakov K. (2010). Massively parallel sequencing for monitoring genetic consistency and quality control of live viral vaccines. Proc Natl Acad Sci USA.

[24] USP. Biological Assay Validation (1033). United States Pharmacop., Rockville, MD; 2010.

[25] Tan C.Y. (2005). RSD and other variability measures of the lognormal distribution. Pharmacopeial Forum.

[26] International Conference on Harmonization of Technical Requirements for Registration of Pharmaceuticals for Human Use. Validation of Analytical Procedures: Text and Methodology Q2(R1); 2005.

[27] Shaw J., Jorba J., Zhao K., Iber J., Chen Q., Adu F. (2018). Dynamics of evolution of poliovirus neutralizing antigenic sites and other capsid functional domains during a large and prolonged outbreak. J Virol.

[28] He Y., Mueller S., Chipman P.R., Bator C.M., Peng X., Bowman V.D. (2003). Complexes of poliovirus serotypes with their common cellular receptor, CD155. J Virol.

