# Supplementary material for: Evaluation and validation of next-generation sequencing to support lot release for a novel type 2 oral poliovirus vaccine
Source: Vaccine X. 2021 Jun 11;8:100102. doi: 10.1016/j.jvacx.2021.100102 (PMC8233139; doi:10.1016/j.jvacx.2021.100102)
Supplement: Supplementary data 1 [file mmc1.docx]

**Supplemental materials from**

***Evaluation and validation of next-generation sequencing to support lot release for a novel type 2 oral poliovirus vaccine***

**Authors and affiliations**:

John O. Konz^1^*, Tim Schofield^2^, Sarah Carlyle^3^, Rahnuma Wahid^1^, Azeem Ansari^1^, Jeroen R.P.M. Strating^4^, Ming Te Yeh^5^, Hasmik Manukyan^6^, Saskia L. Smits^4^, Erman Tritama^7^, Latri Rahmah^7^, Dori Ugiyadi^7^, Saskia L. Smits^4^, Raul Andino^5^, Majid Laassri^6^, Konstantin Chumakov^6^, Andrew Macadam^3^

*To whom correspondence should be addressed: John Konz, Center for Vaccine Innovation and Access, PATH, Seattle, WA, United States of America, jkonz@path.org

^1^Center for Vaccine Innovation and Access, PATH, Seattle, WA, United States

^2^CMC Sciences, LLC, Germantown, Maryland 20876, United States

^3^National Institute for Biological Standards and Control (NIBSC), Hertfordshire, United Kingdom

^4^Viroclinics Biosciences B.V., Rotterdam, the Netherlands

^5^University of California San Francisco, San Francisco, United States

^6^Center for Biologics Evaluation and Research, Food and Drug Administration, Silver Spring, MD, United States

^7^P.T. Bio Farma, Bandung, Indonesia

**RT-PCR methods for measuring viral genome concentration**

To quantify the genome copy (GC) number in each spiked sample (Sabin 2 spiked in nOPV2) a quantitative multiplex one-step RT-PCR (qmosRT-PCR) was used. Briefly, the qmosRT-PCR reactions were prepared in 96-well optical plates in a final volume of 25 μL using 2 μL of RNA of test and control samples (2 μL of DNA-plasmid for reference standards) and QuantiFast Multiplex RT-PCR Kit (QIAGEN, Valencia, CA, USA). The RNAs of Sabin 2 and nOPV2 viruses were used as positive controls, and water was used as negative control. Plasmid containing genome of Sabin 2 virus with known GC number was used as standard reference for extrapolation of GC of Sabin 2 in test samples, and plasmid containing genome of nOPV2 virus with known GC number was used as standard reference for extrapolation of GC number of nOPV2 in test samples.

All control and reference standard samples were run in duplicates, and the test samples were run in six repeats. The specific primer pairs and probes used for each virus were: for Sabin 2, forward primer—Sab2-538F, 5′CGGAACAGGCGGTCGCGAA3′, reverse primer—Sab2-605R, 5′GTAGTCGGTTCCGCCACA3′ and probe—Sab2PrbFAM2, FAM-5′TGACTGGCTTGTCGT3′-ZEN/3IaBkFQ, and for nOPV2, forward primer—nOPV2-538F, 5′TTGAGCAGGCAGCTGCAAC3′, reverse primer— nOPV2-605R, 5′GTAGTCGGTTTCGCCATT3′ and probe— nOPV2PrbYAK2, YAK-5′AGCAGCCAGCCTGT3′-ZEN/3IaBkFQ.

The primers were used at a concentration of 0.8 μM each and the TaqMan probes were used at concentration 25 nM each. The qmosRT-PCR procedure was performed using real-time PCR System ViiA7 (Applied Biosystems, Foster City, CA, USA) at the following thermal cycling conditions: one cycle incubation for 20 min at 50 °C and 5 min at 95 °C, followed by 45 cycles, each consisting of 15 s at 95 °C, 15 s at 55 °C and 30 s at 60 °C.

**Local variant cluster bioinformatics effect**

Noting that the frequencies reported for the Sabin-2-specific positions were lower in regions where differences versus nOPV2-c1 were clustered, we explored whether these differences were artifacts attributable to mapping stringency. In Supplemental Figure 1a, the total reads mapped and the coverage at two positions within domain V (530, 561-3 trinucleotide polymorphism [TNP]) are shown to increase as mapping stringency was reduced. Given that the reduced stringency leads to a greater portion of the Sabin-2-derived reads being mapped against the nOPV2 reference, the reported frequency values for Sabin-2-specific positions increase as stringency declines (Supplemental Figure 1b). Spike-induced variants that were well segregated from other variants (e.g. A814C, T1375A, T5587C) were unaffected by varying the mapping stringency, while variants present in dense clusters that generally appear on common reads (T530C, 561-3 TNP) were sensitive to stringency. As stringency was further reduced, the estimated frequency of spike positions converged to values approximating the expected value. Identification of this artifact induced through use of a Sabin-2 spike were considered in specific aspects of the validation data analysis (specifically which SNPs were included in limit-of-detection estimations).

**Supplemental Figure 1.** Impact of mapping stringency on the mapping of reads derived from the Sabin-2 spike (left panel) and the frequency of spike-induced variants for a sample with an estimated 43% genome ratio spike (right panel). Analysis conducted in Geneious Prime.


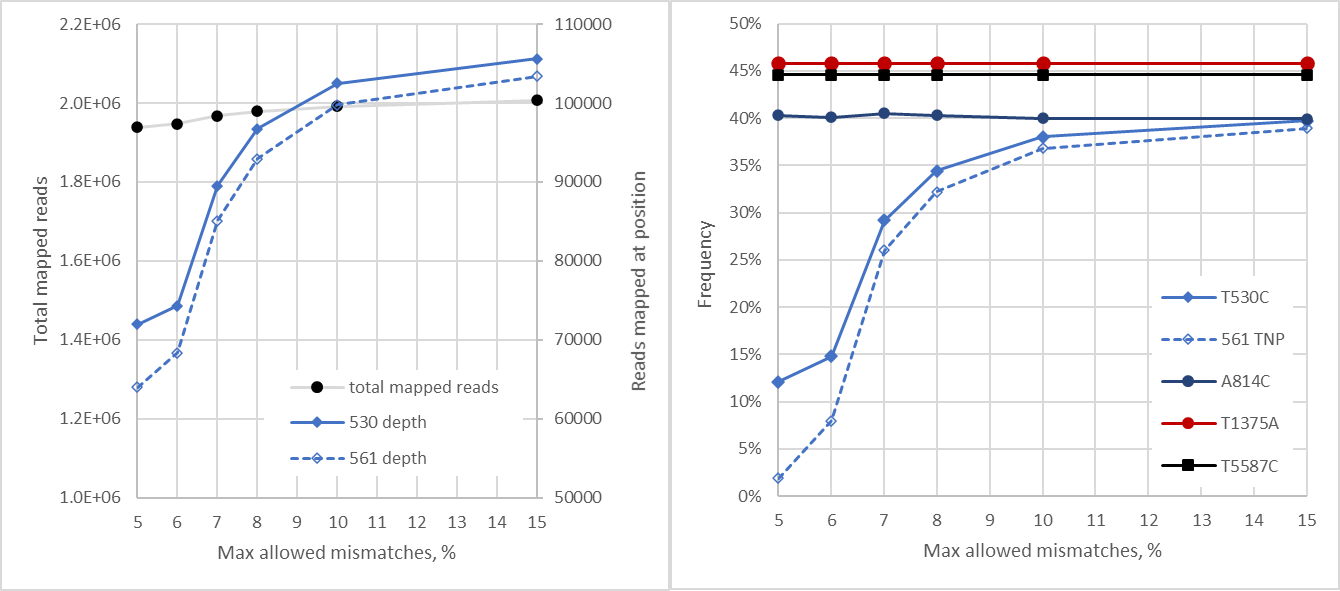


A

B

**Supplemental Information Related to Precision**

Supplemental Figure 2 below shows that for most of the variant frequency estimations, the overall variability was predominantly caused by intra-run variability (repeatability) as opposed to between-run variability.

**Supplemental Figure 2.** Histogram of the fraction of the total error attributable to repeatability. Note that the bin from 1 to 1.1 includes only values of 1.

**Justification of Acceptance Criteria for Intermediate Precision**

The acceptance criteria for intermediate precision “%IP <20% relative standard deviation (RSD, or equivalent with log transformation, for positions 3053 and 3425)” can be justified based upon early data on bulks from the nOPV2 manufacturing process, the specifications on the two positions, and desired process capability (i.e., the ability of the manufacturing process to conform to its specification). The approach described here is a variation of the approach used in USP General Chapter <1033> *Biological Assay Validation*.

The data come from results on positions 3053 and 3425 of 10 bulk lots of nOPV2 (see tables below). Results are expressed as percent of the infectivity (CCID_50_) of Phase 2 lots which showed satisfactory clinical performance. As such the specification becomes >100% for 3425G-containing genomes (coding for parental VP1-295E) relative to a low potency phase 2 lot that showed acceptable immunological response, <100% for A3053G-containing genomes relative to a high potency phase 2 lot that showed acceptable safety.

The geometric mean (GM) across bulk lots was calculated and used to determine the percent intermediate precision that would yield 3-sigma process capability (3-sigma process capability corresponds to an approximate upper bound on proportion of nonconforming lots equal to 0.22%).

The manufacturing variability is the propagation of both assay and process variabilities. The percent of the variability due to assay is accounted for so that the acceptance criterion on precision is not too conservative.

Taking this formulation, the intermediate precision required to achieve 3-sigma process capability is calculated as follows (using the natural log transformation, and assuming a lower specification as for 3425G content):

$$\%IP=100\times\left( exp\left\{ \frac{ln\left( GM \right)-ln\left( 100 \right)}{3\times\sqrt{\%Assay}} \right\}-1 \right)$$

where $\%IP$ is percent intermediate precision, $GM$ is the geometric mean results for the 10 bulk lots, 100 is the lower specification limit, $3$ represents 3-sigma, and $\%Assay$ is the percent of manufacturing variability due to the assay. The formula is similar assuming an upper specification as for 3053G where the numerator becomes $\ln\left( 100 \right)-ln(GM)$.

It is noted that for 3425G content the variability of the assay must be restricted to the acceptance criterion %IP <20% if the manufacturing variability is ≤60% due to assay.

For A3053G the contribution of the assay to manufacturing variability can be as much as 100% with %IP as high as 30% and still yield 3-sigma process capability.

Conclusion: On the assumption that the assay contributes no more than 60% to the observed overall manufacturing variability, an acceptance criterion on intermediate precision %IP<20% will support good process capability.

**Supplemental Table 1**. Measured variability in content of two variants of interest.

| **Lot** | **Content relative to Phase 2 lots (percent CCID_50_)** | |
| --- | --- | --- |
|  | **VP1-N171D (A3053G)** | **VP1-295E (3425G)*** |
| 1 | 36% | 169% |
| 2 | 32% | 174% |
| 3 | 42% | 153% |
| 4 | 54% | 130% |
| 5 | 48% | 143% |
| 6 | 53% | 133% |
| 7 | 29% | 179% |
| 8 | 34% | 169% |
| 9 | 47% | 144% |
| 10 | 46% | 143% |
| **Geometric mean** | **41%** | **153%** |
| **%GCV** | **26%** | **13%** |

*Parental nucleotide and amino acid *i.e.* amount that is not variant.

**Supplemental Table 2**. Requirements for intermediate precision to achieve three sigma capability with different assumed percent contributions of NGS variability to overall observed manufacturing variability.

| **% of Total Variability from NGS** | **% of Total Variability from process** | **Intermediate Precision requirement, % GCV** | |
| --- | --- | --- | --- |
|  |  | **VP1-N171D (A3053G)** | **VP1-295E (3425G)*** |
| 20 | 80 | <30% | <15% |
| 40 | 60 | <34% | <17% |
| 60 | 40 | <40% | <20% |
| 80 | 20 | <51% | <25% |
| 100 | 0 | <79% | <37% |

**Supplemental material – variant co-location analysis**

Comparison of the total variant levels estimated through the co-location analysis and the standard bioinformatic analysis was completed for the pairwise analyses for 15 lots summarized in this study and discussed in the main body of the paper. The concordance of total SNP levels from these analyses with the standard bioinformatic method are shown in Supplemental Figure 3 and support that the subset of the reads able to be utilized for the co-location analysis were representative of the full set of reads used for the standard NGS reporting.

Supplemental Figure 3. Comparison of total variant frequencies estimated from standard bioinformatics using the full genome reference to procedure mapping reads to regions including pairs of variants of interest with full reference coverage required: reference is nucleotide sequence 2969-3054 (panels a and b) and sequence 3052-3426 (panel c)

C

B

A


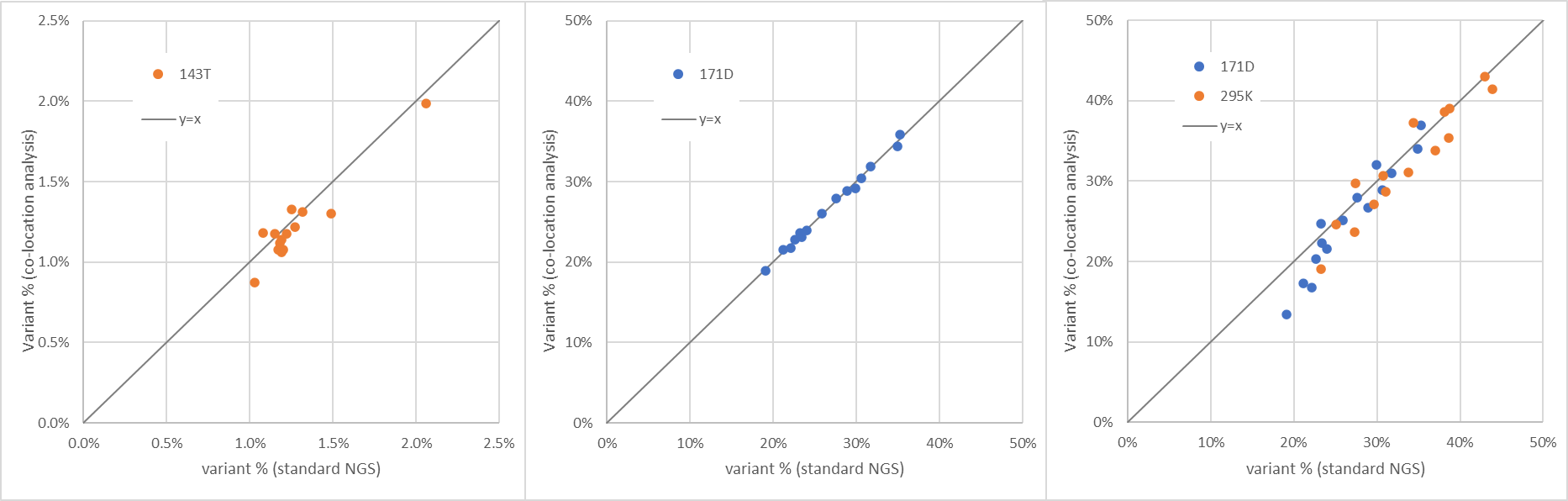


For the nOPV2 lot used in the validation study, a more detailed assessment was conducted. As for the other samples, the paired reads were merged to extend lengths to up to 563 nucleotides. Merged reads were mapped to the full nOPV2-c1 reference and compared to the standard bioinformatic approach to confirm absence of an impact on variant calling (Supplemental Table 3). Next, reads were mapped against the region including all three SNPs (2970, 3053, and 3425) and the 98 reads which covered this full sequence were assessed for the 8 combinations of parental or variant nucleotide in the three positions. The three parental nucleotides were present in 64 reads (65%), representing a higher fraction of parental virus than would be anticipated if the SNPs were randomly distributed (52%). No triple mutants were observed. The single read containing 2970C also contained 3425A (i.e. virus was a 143T 295K double variant). The 3053G, 3425A, and 3053G-3425A double variants were present in 3%, 7%, and 24% of the reads, respectively, suggesting that most 3053G appears with 3425A and the majority of 3425A SNPs appear with 3053G. Because of the limited number of reads mapping across the full region, the same analysis was repeated for smaller regions covering the 2970/3053 and 3053/3425 pairs to assess their colocation in more depth, as summarized in the main text and above. For the 2970/3053 pair over 42,000 reads covered the region, while 843 reads covered the 3053/3425 region. The pairwise results shown in Supplemental Table 4 were similar to the results from the analysis covering the full region, with the 2970C-3053G double mutant being rare, and the majority of both the 3053G and 3425A SNPs appearing together in a double mutant. Over 60% of the 3425A and almost 90% of the 3053G appeared together. In addition, the overall SNP frequencies determined by the approaches that mapped reads to a portion of the genome were reasonably concordant with the results from standard full genome mapping (Supplemental Table 3).

**Supplemental Table 3.** Selected SNP frequencies calculated following different mapping procedures using Geneious

| SNP | Coding impact | Standard analysis | SNP frequencies for different reference sequences, local coverage in parentheses | | | |
| --- | --- | --- | --- | --- | --- | --- |
|  |  |  | Full genome (~66000) | 2969-3426*  (98) | 2969-3054* (42224) | 3052-3426* (843) |
| T2970C | VP1-I143T | 2.1% | 1.9% | 1.0% | 2.0% | - |
| A3053G | VP1-N171D | 22.6% | 22.5% | 26.5% | 22.8% | 20.3% |
| G3425A | VP1-E295K | 31.1% | 31.1% | 31.6% | - | 28.7% |

*Full coverage of the indicated reference required for read mapping

**Supplemental Table 4.** Co-location analyses for (a) 2970 and 3053 positions and (b) 3053 and 3425 positions. Variants are indicated with an asterisk.

| Variant | 2969-3054 reference | | | 3052-3426 reference | | |
| --- | --- | --- | --- | --- | --- | --- |
|  | 2970T (143I) | 2970C (143T)* | Total | 3425G (295E) | 3425A (295K)* | Total |
| 3053A (171N) | 75.2% | 2.0% | 77.1% | 68.9% | 10.7% | 79.6% |
| 3053G (171D)* | 22.8% | 0.03% | 22.8% | 2.3% | 18.0% | 20.3% |
| Total | 97.9% | 2.0% | 99.9% | 71.2% | 28.7% | 99.9% |
